# Influence of oromotor functions on motor development and feeding outcomes in children with cerebral palsy

**DOI:** 10.1080/07853890.2025.2479587

**Published:** 2025-03-19

**Authors:** Amira F. Ibrahim, Sara Y. Elsebahy, Monira I. Aldhahi, Mai M. Khalaf, Ahmed A. Torad, Mona Mohamed Taha, Amira F. El-Sheikh

**Affiliations:** a Department of Physical Therapy for Pediatrics, Faculty of Physical Therapy, Cairo University, Giza, Egypt; b Department of Physical Therapy for Pediatrics, Faculty of Physical Therapy, Kafr El-Sheikh University, Egypt; c Department of Rehabilitation Sciences, College of Health and Rehabilitation Sciences, Princess Nourah bint Abdulrahman University, Riyadh, Saudi Arabia; d Department of Basic sciences for physical therapy, Kafr El-Sheikh University, kafrelsheik, Egypt

**Keywords:** Cerebral palsy, feeding outcomes, motor development, oromotor functions, oral

## Abstract

**Background/Objective(s):**

Children with cerebral palsy often face feeding and swallowing issues, the most common of which include aspiration during feeding and potential pulmonary complications. This impairment can influence other areas of development, including gross motor function, fine motor skills, and oromotor functions involved in feeding and swallowing. This study aimed to investigate the relationship between the level of gross motor function, feeding level, oromotor structural dysfunction, tongue thrust, and eating performance in children with cerebral palsy (CP).

**Patients/materials and methods:**

This cross-sectional study included a total of fifty-five children diagnosed with spastic diplegic cerebral palsy, with a mean age of 4.84  years and a mean weight of 12.46  kg. Participants’ ages ranged from 2 to 14  years. All patients underwent evaluations to determine their level of gross motor function using the Gross Motor Function Classification System (GMFCS), feeding level assessed using the Functional Oral Intake Scale, assessment of oromotor structural dysfunction using the Orofacial Motor Functional Assessment Scale, tongue thrust using the Tongue Thrust Rating Scale, and feeding performance using the Oral Motor Assessment Scale.

**Results:**

Correlational analysis revealed that children with better gross motor function tended to exhibit improved feeding levels, orofacial motor function, and feeding performance (*p* < 0.05); however, no significant relationship was observed between gross motor function and tongue thrust. Regression models showed moderate positive correlations for age, weight, and height, with respective *R*^2^ values of 0.21, 0.23, and 0.28, indicating some influence on outcomes, but with much unexplained variability.

**Conclusions:**

Gross motor function significantly influences feeding and orofacial motor function. These findings suggest that individuals with higher feeding levels tend to have better overall orofacial motor function and feeding performance. Understanding these relationships can inform interventions aimed at improving feeding outcomes and the overall quality of life of children with CP.

## 1. Introduction

Cerebral palsy (CP) is a group of non-progressive neuromuscular illnesses caused by abnormalities in the developing fetal brain. Certain problems are more common or severe in patients with CP than in the general population, although CP does not directly cause any specific oral abnormalities [1]. Children with CP are frequently affected by oral motor difficulties and eating disorders, which can arise up to 90% of the time. Malnutrition, dehydration, missing teeth, inconsistent transitions after puberty, aspiration pneumonia, and mortality are all associated with these issues. Additionally, these issues may halt normal development at crucial junctures. Consequently, it is imperative to identify oral motor and swallowing abnormalities in children at an early age [2].

Inadequate food manipulation, loss of oral food (lack of food retention), growth retardation, low body mass index (BMI), open lips, abnormal tonus, and muscular imbalance of the oral structures [3]. Chronic aspiration, esophagitis, gastroesophageal reflux, respiratory tract infections, and pneumonia are associated with high morbidity rates [4].

Children with CP often experience eating disorders and pulmonary complications [5]. These difficulties arise because synchronized motions of the lips, tongue, cheek, and mandible, which are essential for eating and swallowing, are controlled by the central nervous system [6,7]. Children with CP who have dysphagia find it difficult to eat and swallow only small amounts of nutrients. Maintaining healthy eating habits [8].

The motor development process, particularly during the first year of life, is a fundamental component of the oral sensorimotor system. At the level of gross motor function, this process involves the progressive stabilization of the head and trunk in a seated position, either independently or with external support, while simultaneously facilitating hand-eye coordination to initiate purposeful actions [9]. At the oral level, the motor development process encompasses articulation-related mechanisms, normalization of vagus nerve responses, and the maturation of multidirectional tongue movements. This progression establishes a coordinated relationship through the refinement of motor skills, integrating distal-to-proximal control to facilitate stabilization and sensory integration. These foundational motor patterns support the development of fine movements essential for the precise functions of the speech organs [10].

It is commonly known that difficulties in swallowing and eating prevent people from obtaining sufficient nutrition [11]. The quality of life of a person can also be significantly affected by feeding and swallowing problems [12]. Due to the complex nature of oral feeding, the oral cavity and other bodily systems must be healthy and functioning appropriately. Food is prepared in the oral cavity, generating a bolus that is subsequently thrown back, initiating a swallowing motion that transports the bolus to the stomach. Oral preparation, transition, pharyngeal, and esophageal are other names for the stages of this operation [13]. Pediatricians must assess the oral feeding process to ensure safety and appropriate nutritional intake for healthy growth and development [14]. Gross motor function, which refers to the use of large muscle groups for activities such as walking, running, and maintaining posture, is often impaired in children with CP [15].

This impairment may impact various aspects of development, including fine motor skills and oromotor functions essential for feeding and swallowing. The Gross Motor Function Classification System (GMFCS) was utilized to classify the severity of motor function impairment, offering valuable insights into the associated challenges in feeding and swallowing.

This study aims to examine the relationship between oral motor structural mobility impairments, tongue thrust, motor function, and feeding levels in children with cerebral palsy (CP) diagnosed with dysphagia. The findings will be instrumental in guiding rehabilitation programs, ensuring targeted interventions that facilitate more effective and accelerated improvements in these children. Although previous studies have explored the correlation between gross motor function and oral motor function, there is a paucity of research specifically linking the structural mobility of individual oral motor components to functional and feeding levels. This study seeks to address this research gap by providing a more comprehensive understanding of the interplay between oral motor impairments and feeding difficulties in children with CP.

## 2. Materials and methods

This cross-sectional study was conducted over 6 months, from January 2023 to June 2023. A total of 55 cerebral palsy children, including 31 males and 24 females, were enrolled in the study. To avoid a type II error, a preliminary power analysis [power (1 − α error *P*) = 0.90, α = 0.05, correlation = 0.49] determined a sample size of 43, considering a drop rate of 15% sample size of 55 subjects have been recruited. This correlation was calculated based on a pilot study of 12 subjects, considering that gross motor level was assessed using the Gross Motor Function Classification System (GMFCS) and Orofacial Motor Function Assessment Scale (OFMFAS) as primary variables. The power analysis was carried out using G*Power 3.1.9.2 software, using the exact family and correlation: bivariate normal model. The study procedures were explained to the parents, all of whom signed the consent forms.

The children ranged in age from 2 to 14 years old, with an average age of 4.84. The participants were selected from different Outpatient Clinics (Faculty of Physical Therapy, Cairo University, El Matareya Teaching Hospital, and Kafr El-Zayat General Hospital). Regarding the types of CP, 18 children had spastic diplegia, nine had spastic quadriplegia, 12 had mixed type, five had spastic hemiplegia, 4 had dyskinetic type, 4 had hypotonia, 2 had ataxic type, and 1 had quadriparesis. According to the FOIS, patients had a score of 4–6. According to GMFCS, patients had a score of 2–5. All the patients had problems during the oral swallowing phase.

Children who had oral hypersensitivity, epileptic fits, dysphagia linked to any condition other than cerebral palsy, such as a congenital abnormality, or who had undergone orthopedic or neuromuscular surgery in the head or neck were excluded from the trial. Before the study, interviews were conducted with the children’s parents to explain the purpose and procedures of the study, and they signed a consent form. This study was approved by the ethics committee of the Faculty of Physical Therapy, Kafrelsheikh University (No: 2/2023/32). This study was conducted in accordance with the ethical standards set by the 1964 Declaration of Helsinki, and all participants provided written informed consent.

### 2.1. Procedures

Before beginning the evaluation procedures, the children’s weight (kg) and height (cm) were recorded using a standard weight- and height-measuring scale. The children were evaluated on the day of regular scheduled visits. Each child was evaluated separately in a private calm setting. The evaluation process took approximately 45 min to complete.

The gross motor level was assessed using the GMFCS. It is a multilevel categorization technique that helps describe varying levels of severity in children with CP. Each child performed functional activities without interruption from the caregiver or therapist. The level of activity was recorded according to age. Children at levels I, II, and III were considered to be in the high-functioning CP group, whereas those at levels IV and V were in the low-functioning CP group. [15].

### 2.2. Assessment of orofacial motor function

The Orofacial Motor Functional Assessment Scale was used to evaluate oromotor structural impairment. It is an easy-to-use, accurate, and valid method of assessment, and the subject was positioned in the best sitting position in a ventilated room with the trunk and pelvis aligned while avoiding hip hyperextension. The shoulder girdle was kept forward with abduction of the scapulae, and the cervical spine was elongated [16].

It is intended to assess the strength of the lip muscles (puff out the cheeks/maintain pressure), glossopharyngeal, vagal, and hypoglossal motor activity, as well as rapid coordinated jaw, lip, tongue, and palatal movement by saying ‘AH’. Other voluntary facial movements assessed included opening and closing the mouth, protruding the jaw, and lateral movements. Additionally, aberrant oral reflexes such as sucking, tonic biting, gagging, and rooting were assessed. [17]. The final score was obtained by summing all the sub-item scores. The minimum and maximum scores are 0 and 60, respectively. According to Santos et al. (2005), subjects were classified as severely impaired (score ≤19), moderately impaired (20 < score <31), slightly impaired (32 < score < 41), or very slightly impaired (score ≥ 42) [16].

Eating performance was evaluated using the Oral Motor Assessment Scale. [18]. It is a precise and reliable technique for evaluating the oral motor abilities of children with neurological impairment. Mouth Closure: This evaluation measures an individual’s proficiency in feeding utensils. It focuses on the motor function of the perioral muscles, particularly the orbicularis oris muscle and its marginal and labial sections, which are responsible for lip closure. Lip Closure on Utensil: This assessment examines the motor abilities of the peri-orbicularis muscles, especially the orbicularis Oris muscle and its different parts, with the primary function being the effective closure of the lips around the feeding utensil. Lip Closure During Deglutition: Anterior lip sealing during swallowing is critical for moving the food bolus into the esophagus. This evaluation assesses an individual’s ability to maintain control over the food bolus and swallow without food loss. The expansion of the food bolus indicates a lack of coordinated and efficient movement of the perioral muscles and tongue. Mastication, straw suction, and liquid control during deglutition were the seven components of this scale. The mother was asked to feed the child normally with the following types of food: semi-solid (cake), soft (Yoghurt) served with a spoon, or liquid (served in a cup with straw). A mobile phone video camera was used to record the child’s mastication, suction, and deglutition. After completing the recording, the oral motor skills were scored for each item. The caregiver or child ingested the food without interfering with the way, reinforcing the exclusive observational characteristics of the OMAS. It has a score of 4, ranging from 0 to 3. A score (0) is passive, and a score (1) is sub-functional. Score (2) is semi-functional, and score (3) is functional. [19].

Oral intake was assessed using the Functional Oral Intake Scale (FOIS). It is a standardized clinical measure for detecting feeding progress in children with feeding problems. The FOIS for newborns has demonstrated sufficient validity and reliability, reflecting the growth of oral food in infants. According to our research, this measure may help record infants’ feeding skills and assess how well the interventions are working [20]. This scale may be used in children with tube-dependent or oral diets. It has seven levels, each denoting a different type of oral intake ability, with higher scores indicating a better swallowing function. By asking the mother about the types of food that the child could eat and about the food texture and its consistency, whether it was solid, soft, or mashed. After the mother was asked, I chose the oral intake level from the 4th to the 7th levels.

The scale’s rating scores were categorized as follows: level 1 indicated no oral intake; level 2 involved tube dependence with minimal ingestion of food or liquid; level 3 included tube supplements with regular oral intake of food or liquid; level 4 represented a complete oral diet with a single consistency; level 5 encompassed a complete oral diet with multiple consistencies requiring special preparation; level 6 denoted a complete oral diet with multiple consistencies without special preparation, but limited to specific foods; and level 7 signified a complete oral diet without any restrictions [8].

Tongue thrust movement was assessed using the Tongue Thrust Rating Scale. It is valid, quick, reliable, and easy to use in determining the severity of tongue thrust in children. It has a score of 4, ranging from 0 to 3. A score (0) is no tongue thrust, which is a better scale score, while a score (3) is a severe tongue thrust positioned out of the mouth, which is a worse scale score [21].

### 2.3. Statistical plan

Descriptive statistics were used to summarize the characteristics of the study participants, including their age, weight, and height. Frequency analysis was conducted to examine the distribution of the diagnosis and sex variables, as well as gross motor function level, feeding level, Orofacial Motor function, tongue thrust, and feeding performance. In addition, ranking was used to describe the nonparametric data.

Spearman’s correlation coefficients were calculated to assess the relationships between gross motor function level, feeding level, Orofacial Motor, tongue thrust, and feeding performance, as most of the variables were ordinal variables. Spearman’s rho coefficient values represent the strength and direction of the relationship, while the corresponding p-values indicate the statistical significance of the associations.

SPSS version 25 was used to conduct the statistical analysis. A normality test was conducted using the Shapiro-Wilk test, which revealed that the data were not normally distributed. The Mann–Whitney *U* test, also known as the Wilcoxon rank-sum test, was conducted for all variables between females and males. The level of significance was set at *p* < 0.05. Additionally, a linear regression model was used.

## 3. Results

A total of 55 children with different types of cerebral palsy were included in this study. The mean age of the participants was 4.84 years, with a median age of 4.2 years. The standard deviation (SD) indicated a moderate amount of age variability, with a value of 2.6 years. The mean weight of the individuals was 12.46 Kg, with a median weight of 12 kg. The standard deviation for weight was 3.35 Kg. For height, the mean height of the individuals is 0.98 ± 0.14 m, with a median height of 0.95 m. Of the participants, 24 (43.64%) were female. Additionally, 31 males represented 56.36% of the sample. Table 1 shows the correlations among the variables in the current study.

**Table 1.** Matrix of the correlations between different variables in the study.

Variables	Feeding level		Orofacial motor function		Tongue thrust		Mouth closure		Lip closure on the utensil	
	Spearman’s rho	*p*-value	Spearman’s rho	*p*-value	Spearman’s rho	*p*-value	Spearman’s rho	*p*-value	Spearman’s rho	*p*-value
Gross motor function level	− 0.34	0.01*	− 0.55	<0.001*	0.41	0.002*	− 0.48	<0.001*	−0.46	<0.001*
Feeding level			0.2	0.14	− 0.32	0.01*	0.41	0.002*	0.39	0.003*
Orofacial Motor Function					− 0.17	0.21	0.26	0.05*	0.27	0.04*
tongue thrust							− 0.16	0.23*	−0.19	0.16
Variables	Lip closure during deglutition		Food control during deglutition		Mastication		Sucking Straw		Liquid control during deglutition	
	Spearman’s rho	*p*-value	Spearman’s rho	*p*-value	Spearman’s rho	p-value	Spearman’s rho	p-value	Spearman’s rho	p-value
Gross motor function level	− 0.2	0.14	− 0.46	<0.001*	− 0.32	0.01*	− 0.37	0.006*	− 0.12	0.37
Feeding level	0.28	0.04*	0.51	<0.001*	0.21	0.12	0.35	0.008*	0.28	0.04*
Orofacial motor function	0.24	0.08	0.42	0.001*	0.25	0.06	0.39	0.003*	0.22	0.11
Tongue thrust	− 0.22	0.11	− 0.19	0.15	− 0.38	0.005*	− 0.24	0.08	− 0.34	0.01*

* Denotes *p*-value <0.05.

As shown in Table 2, only 40 cases were included in the comparison between diplegic and quadriplegic children, as 27 were diagnosed with lower limb affection only, while 13 were diagnosed with affection of the four limbs. Table 3 shows the effect of gross motor function on the feeding level and orofacial motor function.

**Table 2.** Comparison between diplegic and quadriplegic patients.

Variables	Mann–Whitney *U*	Wilcoxon W	*p*-value	*N* (Mean Rank) [Sum of ranks]	
				Diplegic	Quadriplegic
Gross motor function level	69	447	0.001*	27(16.56) [447]	13(28.69) [373]
Feeding level	165.5	256.5	0.75	27(20.87) [563.5]	13(19.73) [256.5]
Orofacial motor function	120.5	211.5	0.11	27(22.54) [608.5]	13(16.27) [211.5]
Tongue thrust	158	536	0.59	27(19.85) [536]	13(21.85) [284]
Mouth closure	100	191	0.02*	27(23.3) [629]	13(14.69) [191]
Lip closure on the utensil	129.5	220.5	0.15	27(22.2) [599.5]	13(16.96) [220.5]
Lip deglutition closure	134.5	225.5	0.21	27(22.02) [594.5]	13(17.35) [225.5]
Food control during deglutition	142.5	233.5	0.3	27(21.72) [586.5]	13(17.96) [233.5]
Mastication	117	208	0.07	27(22.67) [612]	13(16) [208]
Sucking Straw	174	265	0.96	27(20.56) [555]	13(20.38) [265]
Liquid control during deglutition	161	539	0.65	27(19.96) [539]	13(21.62) [281]

**Table 3.** Kruskal-Wallis test and mean rank for the effect of gross motor on the feeding level, and orofacial motor functions.

Variables	Median	*H* value	*p*-value	Mean rank of gross motor score			
				2	3	4	5
Feeding level	5	7.53	0.057	41	29.7	27.63	22.76
Orofacial motor function	26	16.62	<0.001*	41	37.55	28.58	17.5
Tongue thrust	1	9.59	0.022*	20	20.6	26.66	36.03
Mouth closure	1	13.31	0.004*	43	32.2	28	20.11
Lip closure on the utensil	1	12.72	0.005*	43	32.05	27.79	20.47
Lip closure during deglutition	2	4.10	0.25	30	29.2	32.18	22.37
Food control during deglutition	3	13.54	0.004*	42	30	30.71	19.26
Mastication	2	5.79	0.122	34	34.95	27.66	22.42
Sucking straw	1	12.78	0.005*	47	28.7	24.89	23.84
Liquid control during deglutition	2	3.10	0.376	37	25.65	26.87	26.97

Regarding gender differences, the mean age of boys was 4.47 years (SD: 1.74), while girls had a slightly higher mean age of 5.13 years (SD: 3.14). Regarding weight, boys had a mean weight of 12.43 units (SD: 3.45), and girls had a similar mean weight of 12.49 units (SD: 3.39). In terms of height, boys have a mean height of 0.97 units (SD: 0.13), while girls have a slightly taller mean height of 0.99 units (SD: 0.16). Among girls, the most common diagnosis was Spastic Diplegic CP, with 8 girls compared to 10 boys. Mixed Dyskinetic-Spastic Diplegic CP was more prevalent among girls, with seven girls than among two boys. On the other hand, Spastic Quadriplegic CP is more common among boys, with eight boys than one girl. Other diagnoses include Spastic Hemiplegic CP, Dyskinetic CP, Hypotonic CP, Mixed Dyskinetic-Spastic Quadriplegic CP, Ataxic CP, Quadriparesis, and Transient Hypotonia, with varying frequencies and percentages among boys and girls. Based on the results of the Shapiro-Wilk test, none of the measured variables appeared to follow a normal distribution. Table 4 summarizes the main findings of the Mann–Whitney *U* test for categorical variables between boys and girls.

**Table 4.** Mann–Whitney *U* test results for categorical variables.

Variable	Value	Girls	Boys	*p*-value	Variable	Value		Girls	Boys	*p*-value
Gross motor function level	2	4(57.1%)	3(42.9%)	0.251	Lip deglutition closure	0	4(30.8%)		9(69.2%)	0.047*
	3	3(30%)	7(70%)			1	4(33.3%)		8(66.7%)	
	4	12(63.2%)	7(36.8%)			2	7(41.2%)		10(58.8%)	
	5	5(26.3%)	14(73.7%)			3	9(69.2%)		4(30.8%)	
Feeding level	4	9(52.9%)	8(47.1%)	0.878	Food control during deglutition	0	1(50%)		1(50%)	0.294
	5	8(33.3%)	16(66.7%)			1	2(25%)		6(75%)	
	6	5(41.7%)	7(58.3%)			2	7(41.2%)		10(58.8%)	
	7	2(100%)	0(0%)			3	14(50%)		14(50%)	
Tongue thrust	0	13(56.5%)	10(43.5%)	0.188	Mastication	0	6(42.9%)		8(57.1%)	0.761
	1	4(50%)	4(50%)			1	4(40%)		6(60%)	
	2	0(0%)	8(100%)			2	10(43.5%)		13(56.5%)	
	3	7(43.8%)	9(56.3%)			3	4(50%)		4(50%)	
Mouth closure	0	9(36%)	16(64%)	0.204	Sucking Straw	0	12(52.2%)		11(47.8%)	0.438
	1	6(46.2%)	7(53.8%)			1	5(41.7%)		7(58.3%)	
	2	3(37.5%)	5(62.5%)			2	2(18.2%)		9(81.8%)	
	3	6(66.7%)	3(33.3%)			3	5(55.6%)		4(44.4%)	
Lip closure on the utensil	0	9(34.6%)	17(65.4%)	0.115	Liquid control during deglutition	0	1(100%)		0(0%)	0.424
	1	2(28.6%)	5(71.4%)			1	6(30%)		14(70%)	
	2	6(60%)	4(40%)			2	12(52.2%)		11(47.8%)	
	3	7(58.3%)	5(41.7%)			3	5(45.5%)		6(54.5%)	

Linear regression model results for age, weight, and height. In the Age Model, age showed a moderately positive correlation (*R* = 0.459) with the dependent variable. This indicates that age has a moderate influence on the outcomes. The *R* Square value of 0.21 suggests that approximately 21% of the variation in the dependent variable can be explained by age alone. However, the low adjusted *R* Square value (0.008) suggests that after considering the number of predictors in the model, the explanatory power is weak.

Weight demonstrated a moderately positive correlation (*R* = 0.478) with the dependent variable. This indicated that weight had a moderate effect on the outcomes. The *R* Square value of 0.229 implies that approximately 23% of the variation in the dependent variable can be accounted for by weight. The adjusted *R* Square value (0.032) showed a slight improvement in explanatory power compared with the Age Model.

In the Height Model, the height exhibited a moderate positive correlation (*R* = 0.525) with the dependent variable. This suggests that height has a moderate impact on the outcomes. The R Square value of 0.276 indicates that approximately 28% of the variance in the dependent variable can be explained by height. The adjusted *R* Square value (0.09) indicates a slight improvement in explanatory power compared to the Weight Model. The study sample consisted of children across different GMFCS levels, with 34.55% classified as Level 5, 34.55% as Level 4, 18.18% as Level 3, and 12.73% as Level 2.

Notably, the *R* Square values in all three models were relatively low, indicating that a significant portion of the variation in the dependent variable remained unexplained. Additionally, the adjusted *R* Square values were small, suggesting that the inclusion of additional predictors may not substantially improve the explanatory power of the models.

## 4. Discussion

Children with CP frequently experience eating and oromotor difficulties; however, most studies do not clearly illustrate how these two issues are related. Therefore, the primary goal of our study was to examine the correlations among feeding level, orofacial motor function, tongue thrust, and eating performance in children with CP. Our findings revealed that children with better gross motor function tended to exhibit improved feeding levels, orofacial motor function, and feeding performance, although no significant relationship was observed between gross motor function and tongue thrust. These results highlight the importance of considering gross motor function when developing interventions aimed at improving feeding outcomes in children with cerebral palsy. This study provides valuable insights that can enhance the quality of life of these children by providing targeted and effective rehabilitation programs.

According to our study’s connections, children with stronger gross motor skills also tend to have better orofacial motor skills, mouth closure, lip closure during utensil use, food management during deglutition, mastication, and sucking straws as opposed to tongue thrust. Children with significant motor disabilities are prone to feeding and nutritional issues. Muscle tone, motor control, independence, and persistence of reflexes may limit the ability to feed [22].

Our results were consistent with those reported in the literature. Sulliver et al. They discovered that children who could not walk or needed assistance were far more likely to have trouble chewing and swallowing foods that had lumps and needed to be mashed or liquidized. Children with generalized severe motor impairments, such as spastic quadriplegia, are more likely to have swallowing problems than children with diplegia [23]. A gastrostomy tube is more likely to be used to feed children with significant motor impairments. Additionally, non-walkers are at a higher risk of choking on solid foods [24]. Additionally, our findings support those of children with CP categorized as GMFCS levels IV and V in the study by Calis et al. They also discovered that children in the GMFCS V were substantially more likely to have more severe dysphagia, need dietary restrictions, employ swallowing techniques, and be dependent on others for eating [25].

Our findings are consistent with those showing that the larger the impairment of gross motor function, the higher the score on the scale, and the greater the orofacial motor impairment, indicating that CP may worsen the impairment of oral motor function and facial function. [16]. Benfer et al. validated our findings. The findings demonstrated that children were less likely to have oropharyngeal dysphagia with a higher gross motor capacity score on the GMFM (the likelihood of oropharyngeal dysphagia decreased by 2–11% for every increase in GMFM-66 score) [26]

Additionally, the primary goal of our study was to show that higher feeding levels are linked to superior orofacial motor function, including mouth closure, lip closure during the use of utensils, and liquid control during deglutition, as opposed to tongue thrust. Our findings are in line with research showing that children with orofacial myofunctional problems such as tongue thrust, reverse swallowing, and abnormal tongue movement have abnormal growth and development, eating issues, attention deficits, behavioral difficulties, and speech disorders. [27].

Swallowing impairment may also result from tongue pushing. The muscles surrounding the tongue may affect how they move and weaken when pushed against the teeth. This may make it difficult to chew and swallow meals [28].

Prado et al. discovered a correlation between oral motor control and chewing and swallowing functions for the stability and speed of the Daido-kinesis parameters, which is consistent with our investigation. [29]. In addition, impaired lip and tongue function may have caused food and liquid loss while eating, resulting in lower swallowing scores in drooled patients. Patients with severe swallowing impairments frequently lose food and liquids [30].

Orofacial dysfunction is linked to a small mouth opening capacity, weak biting power, and dental caries, all of which are reflected in chewing performance. Preschoolers may opt for foods with a soft texture to compensate for their inability to chew properly, or may continuously change their chewing technique to chew the food that is offered to them [31]. That was in agreement with Costa et al. who stated that this population’s ability to develop in all aspects is hampered by a combination of poor nutritional status, oral motor dysfunction, swallowing dysfunction, and inadequate dietary intake [32].

Children with CP frequently overbite significantly, impairing their ability to bite and chew. Jaw instability restricts graded opening and closure, making it difficult to manipulate food in the mouth, compromising bolus formation and making swallowing challenging. Jaw thrusting and retraction may make it difficult to eat from a spoon, drink, hold, manipulate food in the mouth, produce boluses, and swallow safely and effectively. Any feeding strategy must consider and control mouth and jaw movements [33].

Lip control can lead to food and liquid leaks, which reduce food intake, worsen nutrition, and contribute to dehydration. Meal accumulates in the lateral sulci owing to poor cheek tone, inhibiting the creation of a cohesive meal bolus [34].

Children with cerebral palsy (CP) are susceptible to inadequate oral intake owing to various factors, including oromotor dysfunction. Disorders affecting the pharyngeal phase of swallowing are linked to an increased risk of aspiration and communication difficulties, which impede their ability to effectively request food and fluids. In summary, Children with CP often have feeding issues due to oromotor dysfunction and poor postural control. The most frequent reasons (oromotor dysfunction and poor postural control) for feeding difficulties in children and the GMFCS are related. The oromotor dysfunction and postural control worsened with increasing GMFCS weight. Risk factors for eating issues in youngsters include the GMFCS and the type of spastic CP. On believe that malnutrition in children with CP connected to morbidity and mortality can be minimized by identifying feeding challenges in children with CP based on the GMFCS scale [35].

The second goal of our study was to compare gross motor function levels and mouth closure between diplegic and quadriplegic individuals. While other factors were unaffected, there was a statistically significant difference between the diplegic and quadriplegic individuals in these areas. Our study supports the findings of Bangash et al. who found a significant correlation between spastic cerebral palsy and gross motor impairments in GMFCS levels I–V [36]. The presence of motor dysfunction in patients with spastic quadriplegic CP affects their ability to eat because of poor postural control, uncontrollable movements, immobility, inability to self-feed, and seating challenges [6].

The third goal was to evaluate gender differences across all the variables. Although there was no statistically significant difference in the distribution of sexes across the different CP types, dyskinetic CP and spastic bilateral forms, particularly spastic diplegia, were more common in males than in females [37], and ataxic diplegia affected more females. Girls with diplegia performed better in both fine and gross motor abilities in young infants with hemiplegia [38] and in the standing subscale of the GMFM [39].

Age, weight, and height were moderately and positively correlated with promoter function, gross motor function, and feeding level. However, the comparatively low corrected R-squared values suggest that the predictive potential of age, weight, and height is limited. Therefore, additional variables or factors that were excluded from the study may have had a greater impact on promoter function, feeding level, and gross motor function. Age may be a factor Finally, roughly 27.6% of the variance in the dependent variables can be described by height. Approximately 21% of the variance in the variables can be explained by weight, followed by 22.9% of the variance [40].

## 5. Conclusions

Feeding and orofacial motor functions are strongly affected by gross motor function. According to the results, people who eat more frequently typically have stronger orofacial motor skills and better overall feeding performance. Interventions targeted at enhancing feeding outcomes and the general quality of life of children with cerebral palsy can benefit from understanding these linkages.

## Data Availability

The data are available upon request from the corresponding author.
